# Residual Dyslipidemia Leads to Unfavorable Outcomes in Patients with Acute Coronary Syndrome after Percutaneous Coronary Intervention

**DOI:** 10.1155/2016/6175948

**Published:** 2016-01-06

**Authors:** Bin Que, Chunmei Wang, Hui Ai, Xinyong Zhang, Mei Wang, Shaoping Nie

**Affiliations:** Emergency and Critical Care Center, Beijing Anzhen Hospital, Capital Medical University, Beijing Institute of Heart Lung and Blood Vessel Disease, Beijing 100029, China

## Abstract

*Background*. The present study aimed to evaluate the prevalence and prognosis of residual lipid abnormalities in statin-treated acute coronary syndrome (ACS) patients after percutaneous coronary intervention (PCI).* Subjects and Methods*. A total of 3,047 ACS patients who underwent PCI and received statin therapy were included. Plasma concentrations of LDL-C, HDL-C, and TG were measured. For the follow-up study, major adverse cardiovascular cerebrovascular events (MACCE; including total death, cardiovascular death, myocardial infarction, and revascularization) were documented.* Results*. A total of 93.14% of all individuals were followed up for 18.1 months (range, 0–29.3 months). Of all 3,047 patients, those with a suboptimal goal were 67.75%, 85.85%, and 33.64% for LDL-C, HDL-C, and TG levels, respectively. Multiple Cox regression analysis revealed there were significant increases in cumulative MACCE of 41% (HR = 1.41, 95% CI [1.09–1.82], *p* = 0.008), and revascularization of 48% (HR = 1.48, 95% CI [1.10–1.99], *p* = 0.01) in low HDL-C patients with ACS after PCI, but not the high TG group at the end of study.* Conclusions*. Our results showed there is high rate of dyslipidemia in Chinese ACS patients after PCI. Importantly, low HDL-C but not high TG levels are associated with higher MACCE and revascularization rates in ACS patients after PCI.

## 1. Introduction

According to a WHO survey, approximately 4 million people will die of cardiovascular diseases (CVD) in China in 2020. As one of the main risk factors of CVD, dyslipidemia has been widely treated to lower CVD morbidity and mortality. However, accumulating evidence has shown that lipid-lowering treatment with high-intensity statins decreases LDL-C by 20%–30% but only results in 24%–42% reduction of main coronary adverse events [1–3]. In patients treated with statins, residual dyslipidemia occurs when low high-density lipoprotein cholesterol (HDL-C) levels and/or high triglyceride (TG) levels remain. It has been noted that the prevalence of residual dyslipidemia after statin treatment, manifesting as high low-density lipoprotein cholesterol (LDL-C), high TG, or low HDL-C, is high [4–6]. Sirimarco et al. reported that the presence of atherogenic dyslipidemia in subjects with stroke receiving statin therapy was associated with higher residual cardiovascular risk [7]. A cross-sectional trial in China that included 25,697 patients treated with lipid-lowering agents showed that up to 38.5% of patients did not achieve their therapeutic goal. Moreover, 10.4% of very high-risk patients and 11.1% of high-risk patients who attained the LDL-C goal failed to attain non-HDL-C goals [4].

This prospective study was performed to examine the prevalence of residual dyslipidemia in acute coronary syndrome (ACS) patients who underwent percutaneous coronary intervention (PCI) after statin therapy and to evaluate the effect of residual dyslipidemia on major cardiovascular events after 1 year of follow-up.

## 2. Patients and Methods

### 2.1. Patients

Patients who had symptoms of ACS and underwent PCI in Beijing Anzhen hospital (from January 1, 2010, to January 1, 2013) were eligible for this study. Coronary angiography was performed and analyzed to include patients who had either single-vessel disease or multivessel disease. Multivessel disease was defined as ≥50% angiographic diameter stenosis of ≥2 epicardial coronary arteries. Patients with severe congestive heart failure on admission (New York Heart Association III or IV), advanced tumors, or immunologic diseases were excluded. The protocol and consent form were approved by the institutional review board of Beijing Anzhen Hospital. All subjects signed the consent form. Characteristics of all subjects were documented, including age, sex, body weight, height, blood pressure, smoking, and diabetes. All patients enrolled were given optimal medical therapy according to the American Heart Association/American College of Cardiology Foundation “Secondary Prevention and Risk Reduction Therapy for Patients with Coronary and Other Atherosclerotic Vascular Disease” unless contraindicated, including aspirin, anticoagulation if indicated, angiotensin-converting-enzyme inhibitor/angiotensin II receptor blockers, beta-receptor blockers, and statins.

### 2.2. Follow-Up Study

After 3 months of statin treatment, ACS patients were required to attend at outpatient visit to measure their lipid levels. A consecutive series of 3,047 ACS patients treated with statins for at least 3 months were enrolled. Lipid parameters, including total cholesterol (TC), LDL-C, HDL-C, TG, uric acid, creatinine, and high-sensitivity C-reactive protein levels, were measured and collected. The follow-up study was executed by trained personnel using a standardized questionnaire at 6 months and 1 year of follow-up. Major adverse cardiovascular cerebrovascular events (MACCE) were defined as cardiovascular death, reinfarction, revascularization, and stroke. A total of 2,838 (93.14%) patients were successfully followed. A total of 2,639 (99.6%) patients were followed up by phone, 166 patients were followed up in the clinic, and 33 patients were followed up in the hospital.

### 2.3. Statistical Methods

All analyses were performed with Stata, version 11.0 software. Patients who had no lipid parameters were not included in the lipid analyses. Continuous variables were reported using descriptive statistics (mean ± standard deviation [SD] or median with Q1–Q3 interquartile range). For categorical variables, mean ± SD was reported and comparisons were made using the chi-square or Fisher's exact test. Kaplan-Meier analysis was performed to evaluate survival. Prognosis of patients after PCI was analyzed using a Cox proportional hazard model. Results were considered significant or not significant if *p* < 0.05 or ≥0.05, respectively.

## 3. Results

### 3.1. Overall Subject Characteristics

A total of 3,047 patients were enrolled in the present study, and 2,838 (93.14%) had complete follow-up information. The median follow-up time was 543 days; the mean follow-up period was 537 days. The mean age of all 3,047 ACS patients was 59.6 ± 10.6 years, of whom 23.0% were female, 34.1% were smokers, 61.8% had hypertension, and 26.6% had diabetes mellitus.

### 3.2. Residual Dyslipidemia in Patients after PCI

#### 3.2.1. Low HDL-C

The goal of HDL-C level was >40 mg/dL in men and >50 mg/dL in women. Of the 3,047 statin-treated patients, 67.65% had an HDL-C level lower than the goal; among these, 73.1% were male. In the LDL-C goal group, 76.33% of subjects sustained a lower HDL-C than the normal group. The percentage of women in the normal HDL-C level group was lower than that of the normal group (16.5% versus 26.9%, *p* < 0.01).

The percentage of women (16.5% versus 26.9%), age (60.6 ± 10.4 versus 59.1 ± 10.4 years), TC level (189.6 ± 45.5 versus 167.4 ± 40.6 mg/dL), LDL-C level (115.7 ± 37.7 versus 100.8 ± 32.5 mg/dL), TG level (123 [90–171] versus 145 [107–201] mg/dL), glucose level (107.3 ± 35.1 versus 111.4 ± 41.1 mg/dL), percentage of patients with hypertension (58.6% versus 63.8%), and proportion of patients with diabetes (22.5% versus 28.9%) were higher in low HDL-C group than in the normal HDL-C group (*p* < 0.01) (Table 1).

#### 3.2.2. Elevated TG

High TG levels were defined as those >200 mg/dL. Of the statin-treated patients, 42.83% had higher TG levels than the goal, and 76.4% were male. The percentage of women in the normal TG group was similar to the higher TG group (22.7% versus 23.5%, *p* > 0.05). The age (60.8 ± 10.5 versus 58.1 ± 10.6 years), BMI (189.6 ± 45.5 versus 167.4 ± 40.6 mg/dL), TC level (166.3 ± 39.3 versus 187.9 ± 46.4 mg/dL), HDL-C level (0.6 ± 9.1 versus 38.3 ± 9.1 mg/dL), LDL-C level (101.6 ± 33.8 versus 108.7 ± 35.8 mg/dL), glucose level (106.1 ± 36.7 versus 115.2 ± 41.5 mg/dL), estimated glomerular filtration rate (eGFR; 86.6 ± 26.1 versus 84.4 ± 5.2 mL/min), and proportion of patients with diabetes (22.7% versus 31.7%) were significantly different in the higher TG group than in the normal HDL-C group (*p* < 0.01) (Table 1).

#### 3.2.3. LDL-C Goal Attainment

Patients with ACS after PCI belong to the highest cardiovascular disease risk category and have a target LDL-C of <70 mg/dL. Of the 3,047 statin-treated patients, 14.15% had levels lower than the goal; 82.6% of whom were male. LDL-C levels remained higher than goal for 85.85% of subjects, 76% of whom were male.

The two groups were similar regarding age (59.8 ± 10.5 versus 59.6 ± 10.7 years), glucose level (109.7 ± 42.2 versus 109.9 ± 38.5 mg/dL), percentage with hypertension (64.5% versus 61.4%), and proportion with diabetes (30.2% versus 26%). The percentage of women (24% versus 17.4%, *p* < 0.05), eGFR (88.4 ± 31.1 versus 85.4 ± 24.7 mL/min, *p* < 0.05), and proportion of smokers (29.5% versus 34.9%, *p* < 0.05) were higher in the target LDL-C group than in group not meeting the target LDL-C level (Table 1).

### 3.3. The Relationship between Lower HDL-C and Adverse Events

**Figure 1:**
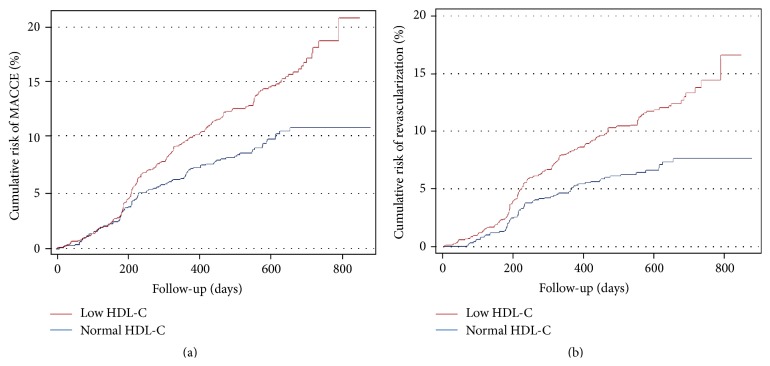
Kaplan-Meier curve of cumulative risk of (a) MACCE (major adverse cardiovascular cerebrovascular events) and (b) revascularization when subjects were grouped according to the HDL-C level.

Multiple Cox regression analysis revealed that there were significant 41% increase in cumulative MACCE (hazard ratio [HR] = 1.41, 95% CI [1.09–1.82], *p* = 0.008) and 48% increase in revascularization (HR = 1.48, 95% CI [1.10–1.99), *p* = 0.01) in lower HDL-C patients with ACS after PCI at the end of follow-up (Figure 1). However, lower HDL-C was not associated with any of the following outcomes: cardiovascular death, total death, myocardial infarction, and stroke (Table 2).

### 3.4. The Relationship between Higher TG and Adverse Events

Between the higher TG group and lower TG group, respectively, the rate of MACCE was 11.9% versus 10.7%, the death rate was 1.8% versus 1.5%, the cardiovascular death rate was 1.5% versus 1.5%, the revascularization rate was 9.1% versus 8.5%, the rate of MI was 0.6% versus 0.4%, and the stroke rate was 1.3% versus 0.8%. Multiple Cox regression analysis revealed that the increase in TG had no relation with any of the following outcomes: MACCE, total death, cardiovascular death, revascularization, MI, or stroke (Table 3).

## 4. Discussion

Reports from the Dyslipidemia International Study have shown that there is a considerable prevalence of residual dyslipidemia after statin therapy worldwide as well as in China [4, 8, 9]. The previous national cross-sectional investigation in China showed that 29.1% of 25,697 patients with statin therapy had no lipid abnormalities, of which 51.2% did not have a TC at goal and 38.5% did not have LDL-C at goal according to 2007 Chinese guidelines [10].

However, our present data showed that the prevalence of residual dyslipidemia is even higher in ACS patients after PCI. There were 63.1% and 85.85% ACS patients after PCI not achieving goal HDL-C and LDL-C levels, respectively, after statin treatment for 3 months (Table 1). Up to 76.33% patients who attained the LDL-C goal failed to achieve the HDL-C goal. Even with a goal LDL-C of <100 mg/dL, only 48.7% patients achieved it, which is much lower than patients in Western countries. In a large cohort of patients hospitalized with CAD, about half have admission LDL levels < 100 mg/dL, while more than half the patients have admission HDL levels < 40 mg/dL and <10% have HDL ≥ 60 mg/dL [11]. A multinational survey that evaluated the proportion of patients achieving LDL-C goals according to relevant national guidelines ranging from 47% to 84% across countries. The overall success rate for LDL-C goal achievement was 73%, but only 67% in high-risk patients. However, only 30% of CAD patients with no fewer than 2 risk factors attained the optional LDL-C goal of <70 mg/dL [9]. It was also reported that 39.6% of the 4,335 statin-treated patients had lipid values within desirable levels in France. LDL-C was not at goal more often (51.8%) in higher-risk patients than in all patients overall (37.2%). Also, high-risk patients with LDL-C not at goal had additional lipid abnormalities (low HDL and/or high TG) more frequently (25.6%) than all patients overall (18.4%) [10].

It has been considered as a risk factor of CAD of low HDL-C level according to a 21-year follow-up study [12]. Moreover, a study enrolling 30,0000 subjects had shown that about 10% of patients with either stroke or transient ischemic attack presenting with residual dyslipidemia (low HDL-C and high TG) had increased cardiovascular risk [7]. Similar with this finding, our present data showed significant increases in cumulative MACCE by 41% and revascularization by 48% in lower HDL-C patients (Table 2 and Figure 1), but not high TG patients (Table 3), with ACS after PCI at the end of follow-up. However, a small-sized case-control study (170 cases and 175 controls) that evaluated the contributions of TG and HDL-C levels in coronary heart disease patients found that high TG and low HDL-C levels contribute strongly and synergistically to CAD after the reduction of LDL-C to the guideline-recommended level [13].

However, our study has limitation that the long-time follow-up studies are still needed to determine if there is truly an association between the low HDL-C level and the worse clinical outcome in ACS patients after PCI. In conclusion, our present study showed a considerably high prevalence of residual dyslipidemia in Chinese ACS patients after PCI. In addition, low HDL-C levels after statin treatment were closely associated with clinical outcomes. Moreover, the results from our present study suggest that more effort need to be made to improve the dyslipidemia situation, not only for LDL-C level but also for HDL-C levels, to get better clinical outcomes in ACS patients after PCI.

## Figures and Tables

**Table 1:** Patient characteristics, risk categories, and lipid parameters.

	All patients	LDL-C at goal	LDL-C not at goal	Normal HDL-C	Low HDL-C	Normal TG	High TG
*N*	3047	431	2616	1127	1920	1742	1305
Age (years)	59.6 ± 10.6	59.8 ± 10.5	59.6 ± 10.7	60.6 ± 10.4	59.1 ± 10.7^##^	60.8 ± 10.5	58.1 ± 10.6^††^
Female (%)	23.0	17.4	24.0^*∗*^	16.5	26.9^##^	22.7	23.5
BMI (kg/m^2^)	25.8 ± 3.1	25.5 ± 3.1	25.9 ± 3.1^*∗*^	25.3 ± 3.0	26.1 ± 3.1	25.4 ± 3.1	26.3 ± 3.0^††^
SBP (mmHg)	128.7 ± 19.3	128.5 ± 19.6	128.7 ± 19.2	129.7 ± 19.1	128.1 ± 19.3^#^	128.8 ± 19.9	128.6 ± 18.3
DBP (mmHg)	78.3 ± 10.9	78.1 ± 10.6	78.4 ± 11.0	78.6 ± 11.0	78.2 ± 10.9	77.9 ± 10.9	79.0 ± 11.0^††^
TC (mg/dL)	175.4 ± 43.8	128.7 ± 22.7	183.2 ± 41.5^*∗∗*^	189.6 ± 45.5	167.4 ± 40.6^##^	166.3 ± 39.3	187.9 ± 46.4^††^
HDL-C (mg/dL)	39.6 ± 9.2	36.2 ± 8.6	40.2 ± 9.2^*∗∗*^	48.2 ± 8.3	34.9 ± 5.5^##^	40.6 ± 9.1	38.3 ± 9.1^††^
LDL-C (mg/dL)	104.6 ± 34.8	60.5 ± 8.4	112.1 ± 32.0^*∗∗*^	111.5 ± 37.7	100.8 ± 32.5^##^	101.6 ± 33.8	108.7 ± 35.8^††^
TG (mg/dL)	137 (100–191)	121 (85–176)	140 (103–193)^*∗∗*^	123 (90–171)	145 (107–201)^##^	105 (84–126)	204 (172–259)^††^
FBG (mg/dL)	109.9 ± 39.1	109.7 ± 42.2	109.9 ± 38.5	107.3 ± 35.1	111.4 ± 41.1^##^	106.1 ± 36.7	115.2 ± 41.5^††^
eGFR (mL/min)	85.7 ± 25.6	88.4 ± 31.1	85.2 ± 24.7^*∗*^	85.6 ± 25.6	85.7 ± 25.8	86.6 ± 26.1	84.4 ± 25.2^†^
LVEF (%)	58.3 ± 10.9	58.1 ± 10.6	58.4 ± 11.0	58.6 ± 11.0	58.2 ± 10.9	57.9 ± 10.9	59.0 ± 11.0
Current smoker (%)	34.1	29.5	34.9^*∗*^	34.1	34.1	33.5	35.0
Hypertension (%)	61.8	64.5	61.4	58.6	63.8^##^	60.4	63.8
Diabetes mellitus (%)	26.6	30.2	26.0	22.5	28.9^##^	22.7	31.7^††^
Multivessel disease (%)	46.7	46.2	46.7	43.7	44.1	46.9	47.0

BMI: body mass index; SBP: systolic blood pressure; DBP: diastolic blood pressure; TC: total cholesterol; HDL-C: high-density lipoprotein cholesterol; LDL-C: low-density lipoprotein cholesterol; TG: triglyceride; FBG: fasting blood glucose; eGFR: estimated glomerular filtration rate; LVEF: left ventricular ejection fraction. ^*∗*^ *p* < 0.05, ^*∗∗*^ *p* < 0.01 compared with LDL-C at goal group; ^#^ *p* < 0.05, ^##^ *p* < 0.01 compared with normal HDL-C group; ^†^ *p* < 0.05, ^††^ *p* < 0.01 compared with normal TG group.

**Table 2:** The comparison of MACCE between normal HDL-C group and low HDL-C group.

	Normal HDL-C		Low HDL-C		Risk ratio (HR)	*p* value
	(*N* = 1127)		(*N* = 1920)			
	*n*	(%)	*n*	(%)		
MACCE	97	8.6	251	13.1	1.41 (1.09–1.82)	0.008^*∗*^
All cause death	19	1.7	32	1.7	1.25 (0.64–2.41)	0.50
Cardiovascular death	19	1.7	27	1.4	1.50 (0.62–3.64)	0.372
Revascularization	68	6.0	201	10.5	1.48 (1.10–1.99)	0.01^*∗*^
Myocardial infarction	5	0.4	11	0.6	0.87 (0.28–2.70)	0.80
Stroke	11	1.0	21	1.1	1.44 (0.65–3.18)	0.37

MACCE: major adverse cardiovascular cerebrovascular events. ^*∗*^ *p* < 0.05 compared with Normal HDL-C group.

**Table 3:** The comparison of cardiovascular events between normal TG group and elevated TG group.

	Normal TG		Higher TG		Risk ratio (HR)	*p* value
	(*N* = 1742)		(*N* = 1305)			
	*n*	(%)	*n*	(%)		
MACCE	208	11.9	140	10.7	0.88 (0.70–1.12)	0.35
All cause death	32	1.8	19	1.5	0.80 (0.42–1.54)	0.51
Cardiovascular death	27	1.5	19	1.5	1.12 (0.52–2.45)	0.76
Revascularization	158	9.1	111	8.5	1.05 (0.81–1.36)	0.69
Myocardial infarction	11	0.6	5	0.4	0.83 (0.27–2.51)	0.74
Stroke	22	1.3	10	0.8	0.63 (0.29–1.40)	0.26

MACCE: major adverse cardiovascular cerebrovascular events.
